# A Fault Diagnosis Approach for Rolling Bearing Integrated SGMD, IMSDE and Multiclass Relevance Vector Machine

**DOI:** 10.3390/s20154352

**Published:** 2020-08-04

**Authors:** Xiaoan Yan, Ying Liu, Minping Jia

**Affiliations:** 1 School of Mechatronics Engineering, Nanjing Forestry University, Nanjing 210037, China; liuying@njfu.edu.cn; 2 School of Mechanical Engineering, Southeast University, Nanjing 211189, China; mpjia@seu.edu.cn

**Keywords:** symplectic geometry mode decomposition, improved multiscale symbolic dynamic entropy, multiclass relevance vector machine, rolling bearing, fault diagnosis

## Abstract

The vibration signal induced by bearing local fault has strong nonstationary and nonlinear property, which indicates that the conventional methods are difficult to recognize bearing fault patterns effectively. Hence, to obtain an efficient diagnosis result, the paper proposes an intelligent fault diagnosis approach for rolling bearing integrated symplectic geometry mode decomposition (SGMD), improved multiscale symbolic dynamic entropy (IMSDE) and multiclass relevance vector machine (MRVM). Firstly, SGMD is employed to decompose the original bearing vibration signal into several symplectic geometry components (SGC), which is aimed at reconstructing the original bearing vibration signal and achieving the purpose of noise reduction. Secondly, the bat algorithm (BA)-based optimized IMSDE is presented to evaluate the complexity of reconstruction signal and extract bearing fault features, which can solve the problems of missing of partial fault information existing in the original multiscale symbolic dynamic entropy (MSDE). Finally, IMSDE-based bearing fault features are fed to MRVM for achieving the identification of bearing fault categories. The validity of the proposed method is verified by the experimental and contrastive analysis. The results show that our approach can precisely identify different fault patterns of rolling bearings. Moreover, our approach can achieve higher recognition accuracy than several existing methods involved in this paper. This study provides a new research idea for improvement of bearing fault identification.

## 1. Introduction

Rolling bearing is one of the most widely used parts of rotating machinery equipment, and also one of the most vulnerable parts. Relevant studies show that more than 45% of rotating machinery equipment faults are caused by bearing damage [1]. Bearing fault signal generated in practice usually has distribution characteristics of nonlinear, non-stationary and non-Gaussian, which indicates that traditional linear analysis method cannot meet the requirement of modern bearing fault diagnosis [2,3,4]. In other words, it is urgent and meaningful to explore a new and high-efficiency nonlinearity bearing fault diagnosis technology.

On the one hand, the common nonlinear bearing fault diagnosis approaches include fractal dimension [5], wavelet transform (WT) [6], sample entropy (SE) [7], permutation entropy (PE) [8] and fuzzy entropy (FE). However, the above methods are all single scale analysis methods based on time series, which means that nonlinear dynamic behaviors and complexity of the original signal cannot be effectively revealed by using the above single scale methods [9]. Given these facts, various multiscale analysis methods are proposed consecutively to describe the complexity and uncertainty of signals at multiple scales, which can achieve richer and more comprehensive bearing fault information. The most common multiscale analysis methods include multiscale Lempel–Ziv complexity (MLZC) [10], multiscale sample entropy (MSE) [11], multiscale permutation entropy (MPE) [12] and multiscale fuzzy entropy (MFE) [13]. However, these methods also have some disadvantages. For instance, anti-interference ability and stability of MLZC need to be improved, especially for complex nonlinear signals. MSE has poor real-time performance and slow computing speed for long time series, and it is not very good for dealing with abrupt signals. Compared with MSE, the calculation time of MPE is improved, but it does not consider the magnitude relationship between amplitudes. MFE is defined based on the fuzziness between signals, and the information described is not clear enough. Besides, similar to MSE, calculation time of MFE is also relatively slow, especially for long data time series. Given these problems, multiscale symbolic dynamic entropy (MSDE) is proposed by Li et al. [14] to extract bearing fault information at multiple time scales. However, due to the application of traditional coarse-grained procedure, MSDE suffers from the problem of missing of partial fault information. To address this issue, in this paper, an improved multiscale symbolic dynamic entropy (IMSDE) is presented to describe dynamic behavior of signal and extract fault features across multiple scales by modifying coarse-grained procedure. Meanwhile, an optimization method named bat algorithm (BA) [15] is employed to determine automatically the key parameters of IMSDE. Compared with some other optimizers (e.g., particle swarm optimization (PSO) [16], genetic algorithm (GA) [17] and ant colony algorithm (ACO) [18]), BA is much better in terms of accuracy and effectiveness of parameter optimization, and has fewer parameters to be adjusted; thus, it is suitable for solving various parameter optimization problems.

On the other hand, due to actual bearing vibration signal contains many noise interferences, which indicates that the results of direct multiscale analysis for the original signal will be affected to some extent. Hence, to remove the noise interferences and improve fault feature extraction ability, it is very necessary to adopt a signal decomposition method to preprocess the original signal. Current popular signal decomposition methods include ensemble empirical mode decomposition (EEMD) [19], empirical wavelet transform (EWT) [20], ensemble intrinsic time-scale decomposition (EITD) [21], variational mode decomposition (VMD) [22], singular spectrum decomposition (SSD) [23] and so on. However, these algorithms also have some disadvantages. EEMD can eliminate modal aliasing by continuously adding white noise to the original signal, but its computation time is very slow for long time series. EWT is not self-adapting in the division of frequency band, and it is dependent on expert experience. ITD suffers from curve deformation and distortion in signal decomposition, because of the fitting of the linear transformation. VMD easily appears over decomposition or under decomposition if the parameter setting is unreasonable. As with the traditional empirical mode decomposition (EMD), there are endpoint effects and modal aliasing in SSD. Given these facts, this paper employs a novel signal processing method named symplectic geometry mode decomposition (SGMD) [24] to improve decomposition performance of nonlinear and non-stationary signal.

Obviously, after signal decomposition and feature extraction, an efficient classifier is required for intelligent fault identification. Typical classifiers contain back-propagation neural network (BPNN) [25], extreme learning machine (ELM) [26] and support vector machine (SVM) [27] and so on. Although these classifiers have made good progress in fault diagnosis, they also have some limitations. For example, BPNN has an overfitting problem and is prone to local optimality. ELM is essentially a single hidden layer neural network with slow convergence and unstable training process. SVM has the advantages of good generalization ability and handling small sample problems, but it also has a large classification error, high computational complexity and no self-adaptability in parameter selection. Faced with these problems, inspired by Bayesian theory, the concept of relevance vector machine (RVM) is proposed by Tipping [28] in 2001, which has been successfully applied in the field of fault diagnosis. Theoretically speaking, compared with SVM, RVM can more effectively output the probability of classified samples with less calculation time, which is suitable for online fault diagnosis [29]. Nevertheless, RVM can only solve two-category identification problem, whereas fault patterns of practical bearing are usually multi-classification problems. On account of this, Psorakis et al. [30] proposed a multiclass relevance vector machine (MRVM), which not only has the advantages of RVM, but also can quickly and directly output the diagnosis probability of each fault patterns. Hence, in the classification layer of the proposed algorithm, this paper selects MRVM as an automatic recognizer to detect effectively different health conditions of rolling bearing. The point to emphasize here is that the deep learning is also widely used in intelligent fault identification of rolling bearing and has obtained many achievements, such as deep belief networks (DBN) [31], sparse auto-encoder (SAE) [32], convolutional neural network (CNN) [33] and generative adversarial networks (GAN) [34] etc., which indicate that deep learning is a hot research direction at present and is worth investing. For this aspect of content, we will conduct further discussion and research in future work.

Summing up the above, the focus of this article is to develop an effective rolling bearing intelligent fault diagnosis method based on SGMD, IMSDE and MRVM, where three tools (i.e., SGMD, IMSDE and MRVM) are, respectively, applied to achieve its three main procedures (i.e., signal preprocessing, fault feature extraction and fault pattern recognition). Main contributions and novelty of this paper are demonstrated as follows:

(1) A new method called SGMD is employed for preprocessing of bearing vibration data, which can obtain a superior noise reduction effect via signal decomposition and reconstruction.
(2) The BA-based optimized IMSDE is proposed to extract bearing fault features at multiscale scales, which can not only overcome the shortcomings of MSDE in description of signal complexity, but also avoid the dependence of parameter selection of MSDE on expert experience.
(3) MRVM is introduced for automatic identification of bearing fault patterns, which has fewer parameters than other classifiers (e.g., BPNN and SVM).
(4) The efficacy and superiority of the proposed method is verified by the experimental investigation and comparative analysis of various methods.

The rest of this paper is structured as following: Section 2 introduces the detailed procedure and flowchart of the proposed method. Meanwhile, in Section 2, some simulation analysis is given to show the efficacy of SGMD and IMSDE. Section 3 conducts the experimental study to validate the effectiveness and superiority of the proposed method for bearing fault identification. Conclusions and discussions are placed in Section 4. Appendix A gives the presentation of the related methods (i.e., SGMD, IMSDE and MRVM).

## 2. The Proposed Method

To effectively identify bearing health condition and improve fault identification accuracy, this section proposes an integrated approach for intelligent fault identification of rolling bearing based on SGMD, IMSDE and MRVM, which is mainly composed of three steps (i.e., signal preprocessing, fault feature extraction and fault pattern recognition). Detailed procedure of the proposed method in this paper is described below.

### 2.1. Signal Preprocessing

**Figure 1:**
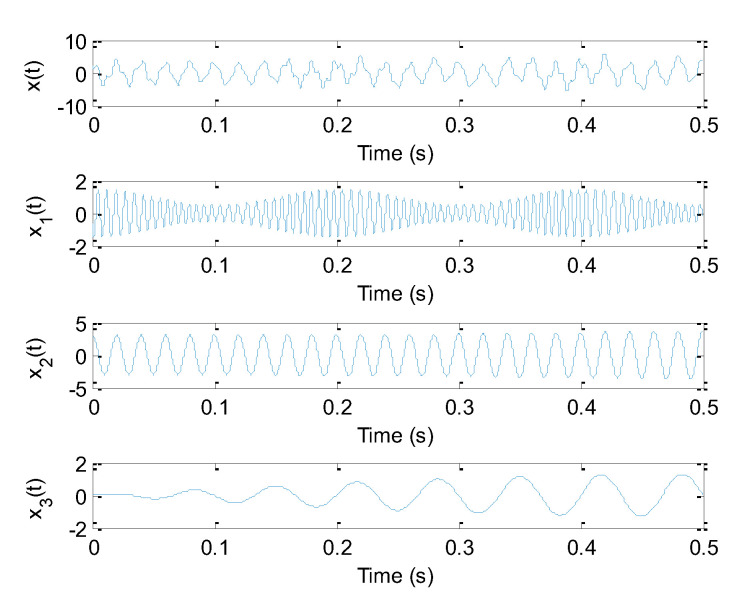
Waveform of the simulated signal and its components.

**Figure 2:**
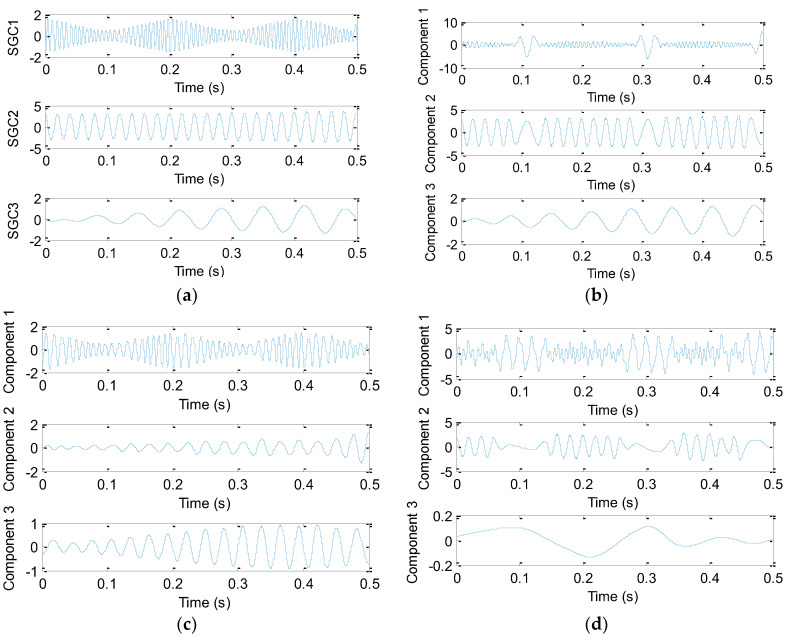
Decomposition results obtained by different methods: (**a**) symplectic geometry mode decomposition (SGMD), (**b**) ensemble empirical mode decomposition (EEMD), (**c**) empirical wavelet transform (EWT) and (**d**) ensemble intrinsic time-scale decomposition (EITD).

When rolling bearing appears a local fault, feature information related to bearing fault is usually submerged in background noise and other interference signals, which indicates that it is very necessary to adopt an effective method to preprocess the original bearing vibration signal, which is aimed at removing the noise and highlighting the fault features. SGMD is a novel adaptive signal processing method, which has a promising application in noise reduction. Therefore, in the first step of the proposed method, SGMD is firstly applied to decompose the collected bearing vibration signal to a sequence of symplectic geometric components (SGC) through several steps (e.g., phase space reconstruction, QR decomposition of symplectic orthogonal matrix and diagonal average operation). Subsequently, the first several SGC components containing main fault information are selected to reconstruct the original bearing vibration signal for the purpose of noise reduction. In this step, SGMD theory is briefly summarized as [24]:

Firstly, phase space reconstruction of the given signal is conducted to obtain a trajectory matrix which can be used to construct Hamiltonian matrix. Secondly, symplectic matrix similarity transformation of the trajectory matrix is calculated to solve the eigenvalues of Hamiltonian matrix. Finally, the eigenvectors corresponding to those eigenvalues are used to reconstruct symplectic geometric component (SGC) containing inherent modulated oscillations. More details about SGMD can be found in Appendix A. To investigate the decomposition performance of SGMD, here, a multi-component amplitude modulation (AM) and frequency modulation (FM) signal *x*(*t*) is simulated by

(1) $$\left\{ \begin{array}{l} x(t)=x_{1}(t)+x_{2}(t)+x_{3}(t)\\ x_{1}(t)=(1+0.5\cos(10\pi t))\cos(300\pi t+2\cos(20\pi t))\\ x_{2}(t)=3(t^{2}+1)\cos(100\pi t)\\ x_{3}(t)=1.3\sin(\pi t)\sin(30\pi t) \end{array}\right.$$

where *x*_1_(*t*), *x*_2_(*t*) and *x*_3_(*t*) are three mono-components of the original signal *x*(*t*), whose main frequencies are 150 Hz, 50 Hz and 15 Hz, respectively. Besides, t∈(0,0.5), that is, the sampling frequency and sampling number, are 8192 Hz and 4096 points, respectively. Figure 1 shows waveform of the simulated signal and its components. Figure 2a–d display the decomposition results obtained using different signal decomposition methods (SGMD, EEMD, EWT and EITD), respectively. Seen from Figure 2a, three components of the original signal *x*(*t*) are perfectly revealed, which are corresponding to 150 Hz, 50 Hz and 15 Hz, respectively. This indicates that SGMD is effective in multi-component signal analysis. As seen from Figure 2b, in the first two components obtained by EEMD there exists a mode mixing problem, which is not consistent with the actual component. Similarly, due to the deviation of amplitude of the second and third decomposed components obtained by EWT, three real ingredients of the original signal *x*(*t*) cannot be accurately extracted in Figure 2c. Besides, in Figure 2d, the decomposition results obtained by EITD are unsatisfactory due to the decomposed waveforms far away from the real waveform. Therefore, from the decomposition results, SGMD can achieve better decomposition performance than other several methods (EEMD, EWT and EITD).

To quantitatively compare decomposition effect of various approaches, several acknowledged metrics (e.g., cross-correlation coefficient ρ, root mean square error (RMSE) and computing time) are calculated in the decomposition results of each algorithm. Table 1 lists the detailed calculation results. It is very obvious in Table 1 that cross-correlation coefficient ($\rho_{1}$, $\rho_{2}$ and $\rho_{3}$) between three components obtained by SGMD and the real components respectively is 0.9987, 0.9998 and 0.9942, which are greater than those obtained by other methods. Besides, RMSE of decomposition results obtained by SGMD is less than those in other methods. In terms of running efficiency, computing time of EEMD is the highest, SGMD is the second largest, but computing time of SGMD is very close to that of EWT and EITD. Hence, in terms of quantitative comparison, compared with other methods, SGMD also shows great advantages, which further proves the effectiveness and superiority of SGMD in multi-component signal decomposition.

### 2.2. Fault Feature Extraction

After using SGMD to preprocess the original bearing signal, it is necessary to adopt an effective method to extract fault features of rolling bearings. According to [14], MSDE with coarse-grained operation is considered as an effective fault feature extraction method and has been successfully applied in bearing fault diagnosis field, which can describe the complexity and uncertainty of nonstationary vibration signal at different time scales. However, in MSDE, traditional multiscale coarse-grained procedure has the problem of missing of partial fault information. Hence, to solve this problem, this section proposes an improved multiscale symbolic dynamic entropy (IMSDE) for fault feature extraction through modifying traditional multiscale coarse-grained procedure with data sliding operation. Description of IMSDE theory can be clearly found in Appendix A.

**Figure 3:**
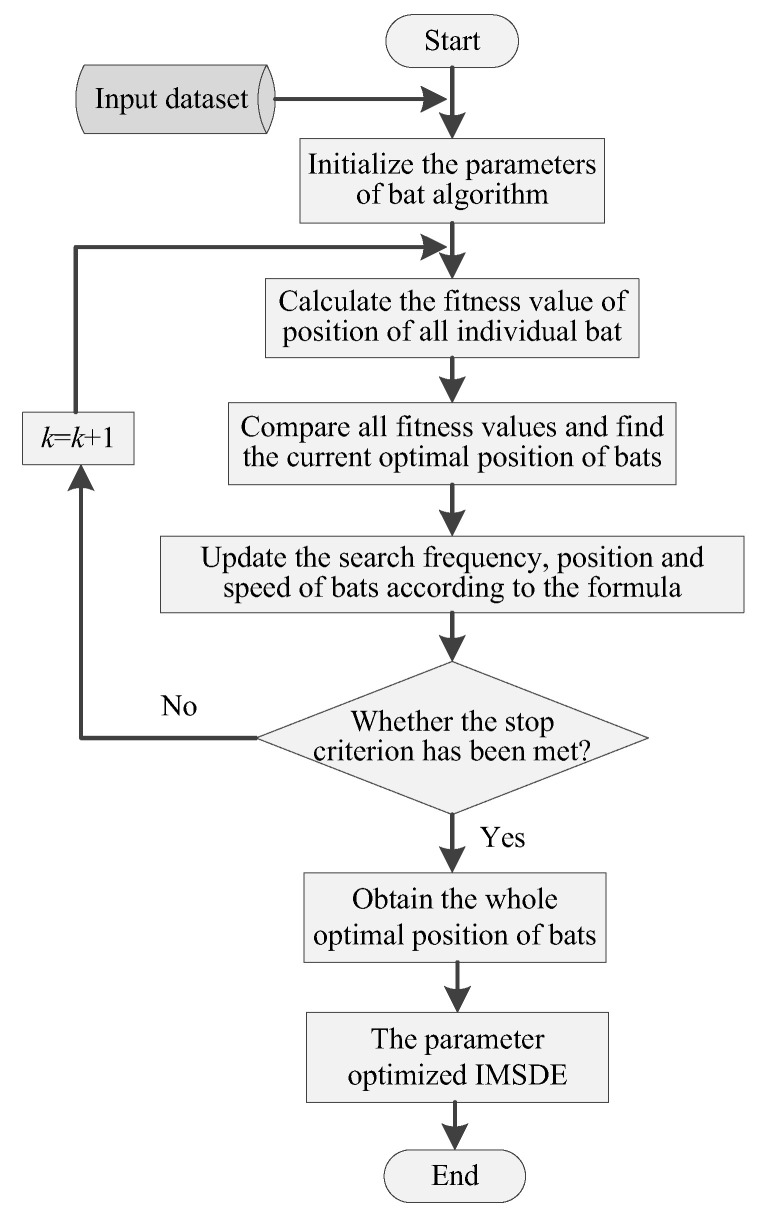
Flowchart of bat algorithm (BA)-based optimized improved multiscale symbolic dynamic entropy (IMSDE).

Specifically, in the second step of the proposed method, IMSDE of the reconstructed bearing vibration data is calculated to construct fault characteristic matrix, where an optimization tool named bat algorithm (BA) is used to optimize the key parameters (i.e., the scale factor τ, embedding dimension *m*, the time delay λ, the number of symbol ε) of IMSDE. By doing this, the optimized IMSDE can avoid the problem of empirically selecting parameters in MSDE and IMSDE. Figure 3 shows the flowchart of the optimized IMSDE. Specific procedure of using BA to optimize the parameters of IMSDE is expressed as follows:

(1) Initialize the parameters of BA. Specifically, set maximum number of iterations *M* = 50, set the population number *N* = 1000, search frequency range $\left[f_{\min},f_{\max}\right]$, speed $V_{i}$ and position $X_{i}$ of bats. Due to the four parameters (τ, *m*, λ, ε) of IMSDE need to be optimized, so the position of each bats is defined as $X_{i}=\left[x_{\tau },x_{m},x_{\lambda },x_{\varepsilon }\right],\;i=1,2,\cdots ,N$, where $x_{\tau }$, $x_{m}$, $x_{\lambda }$, and $x_{\varepsilon }$ are the scale factor, embedding dimension, time delay and the number of symbol, respectively. The upper and lower limits of $X_{i}$ are [2,1,1,2] and [20,6,6,15], respectively.
(2) Define the fitness function of BA as the accuracy rate between the correctly classified samples and total samples, calculate and compare the fitness value of position of all bats, find the current optimal position of bats according to maximum fitness function.
(3) Update search frequency, speed and position of bats according to the following formula:
  (2) $$\left\{ \begin{array}{l} f_{i}=f_{\min}+(f_{\max}-f_{\min})\times \beta \\ V_{i}^{t}=V_{i}^{t-1}+(X_{i}^{t}-X^{*})f_{i}\\ X_{i}^{t}=X_{i}^{t-1}+V_{i}^{t} \end{array}\right.$$
  where β∈[0,1] is a random variable satisfying uniform distribution, $X^{*}$ represents the current global optimal position of bats, and the search frequency $f_{i}$ assigned to each bat needs to satisfy the uniform distribution of $\left[f_{\min},f_{\max}\right]$.
(4) Determine if the iteration stopping criterion has been met. If the stopping criterion is reached, we will obtain the overall optimal position (i.e., the optimal parameters of IMSDE) of bats. Otherwise, repeat step 2 and 3 until the stop criterion is met. Finally, the parameter optimized IMSDE is used for fault feature extraction.

**Figure 4:**
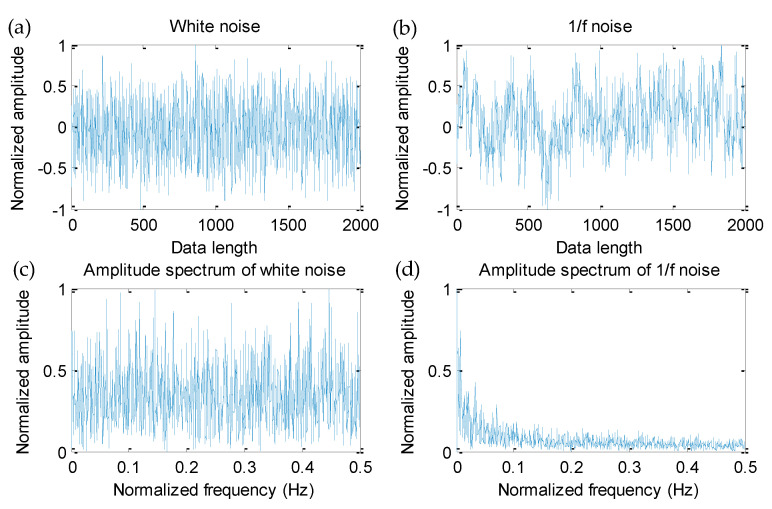
Waveform and amplitude spectrum of two noise signals: (**a**) waveform of white noise, (**b**) waveform of 1/f noise, (**c**) amplitude spectrum of white noise, (**d**) amplitude spectrum of 1/f noise.

**Figure 5:**
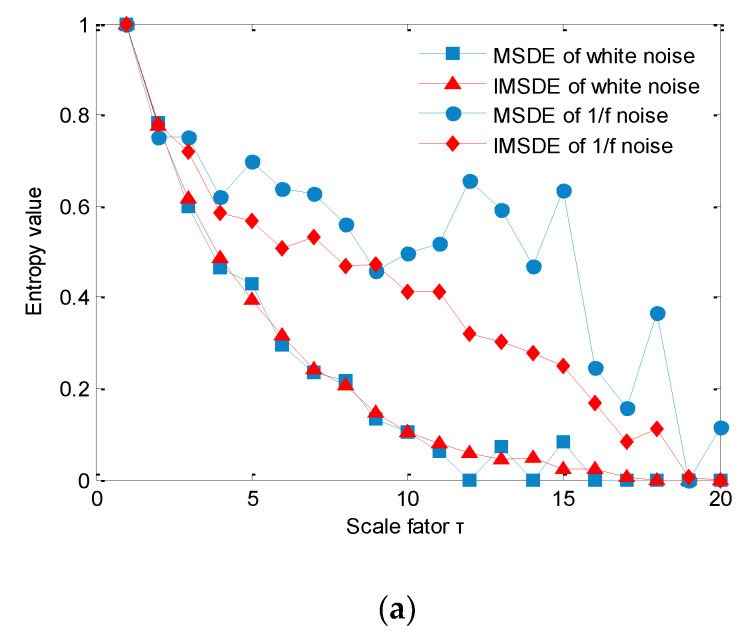

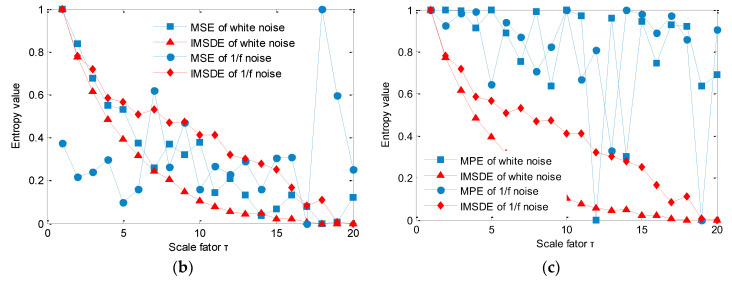
Comparison of multiscale entropy: (**a**) multiscale symbolic dynamic entropy (MSDE) and improved multiscale symbolic dynamic entropy (IMSDE), (**b**) multiscale sample entropy (MSE) and improved multiscale symbolic dynamic entropy (IMSDE), (**c**) multiscale permutation entropy (MPE) and improved multiscale symbolic dynamic entropy (IMSDE).

In this step, to show the effectiveness of the proposed IMSDE and compare the performance of different multiscale entropies in describing complexity of signal, here, two noise signals (white noise and 1/f noise) with a data length of 2000 points are considered. Figure 4 shows time-domain waveform and amplitude spectrum of two noise signals (white noise and 1/f noise). Seen from Figure 4, amplitude of two noise signals fluctuates with the increase of data length and normalized frequency, which indicates that their complexity and irregularity are different. That is, entropy value of two noise signals (white noise and 1/f noise) is discrepant. To describe this difference more clearly, four multiscale entropies (i.e., IMSDE, MSDE, MSE and MPE) of two noise signals calculated. In this comparison, according to the [14], the important parameters of IMSDE are manually selected as τ = 20, *m* = 3, λ = 1 and ε = 12, where τ is the scale factor, *m* is the embedding dimension, λ is the time delay and ε is the symbolic number. Besides, to ensure a fair comparison, key parameters of other multiscale entropies (i.e., MSDE, MSE and MPE) are the same as IMSDE. Figure 5a–c plots the normalized comparison results between IMSDE and other three multiscale entropies (i.e., MSDE, MSE and MPE), respectively. It can be seen clearly in Figure 5 that entropy value of two noise signals obtained by IMSDE is relatively smooth and decreases monotonically as the scale factor increases, which conforms to the rules of complexity variation of noise signal. However, the entropy value of two noise signals obtained by other methods (MSDE, MSE and MPE) is very irregular and fluctuates a lot across the whole scales. In other words, measuring of complexity and regularity signal using other contrastive entropies is not ideal. Hence, the comparison results show the effectiveness of IMSDE in signal complexity assessment and fault feature extraction.

**Figure 6:**
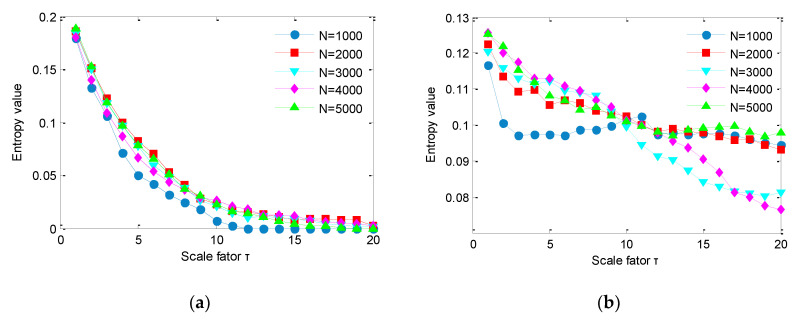
IMSDE of two noises for different data length: (**a**) IMSDE of white noise and (**b**) IMSDE of 1/f noise.

To investigate the influence of data length on IMSDE, we calculate IMDE of two noises under different data length and the results are shown in Figure 6. As seen from Figure 6, for different data lengths, IMDE of two noises has a consistent trend and decreases as the scale factor increases. However, in practical application, the data length is not suitable for settings too large or too small. If data length is too small, estimation of signal complexity is not accurate and the entropy value fluctuates greatly. On the contrary, if data length is too large, more accurate estimation of signal complexity is usually obtained, but it will also take more time to calculate the IMSDE. Thus, data length *N* = 2000 is usually sufficient in using IMSDE to estimate signal complexity and extract fault features.

**Figure 7:**
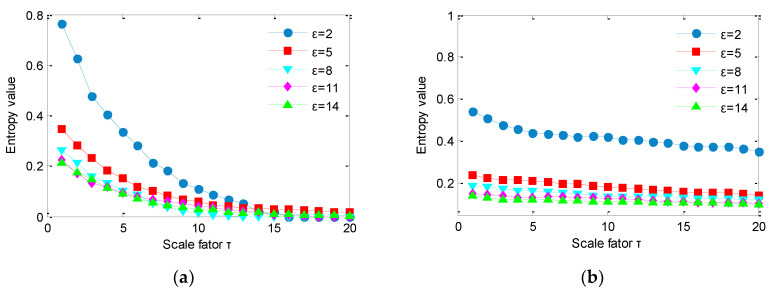
IMSDE of two noises for different symbols: (**a**) IMSDE of white noise and (**b**) IMSDE of 1/f noise.

According to the literature [14], the performance of MSDE is lesser affected by the embedding dimension and time delay than the number of symbols. Thus, here we only investigate the influence of number of symbols on IMSDE. Specifically, we calculate IMSDE of two noises under different number of symbols and the calculation results are plotted in Figure 7. As shown in Figure 7, for the same scale factor, entropy value obtained by IMSDE decreases with the increase of symbolic number. Besides, as the scale factor increases, entropy value obtained by IMSDE also has a downward trend, which indicates that number of symbols has a certain influence on complexity assessment performance of IMSDE, that is, it cannot be set too large or too small. If the number of symbols is too small, feature extraction ability of IMSDE may not be strong. On the contrary, if the number of symbols is too large, calculation of IMSDE will take a long time. Therefore, to make a trade-off between calculation efficiency and feature extraction performance, the number of symbols is usually selected to be from 5 to 12 for practical application.

### 2.3. Fault Pattern Recognition

According to the routine diagnostic procedures, after bearing vibration signal preprocessing and fault feature extraction, an automatic identification process needs to be added in the proposed method. MRVM is a useful fault classification model, which has been proven to have advantages over standard support vector machines and neural networks. Hence, in the last step of the proposed method, MRVM classifier is employed to achieve automatically fault pattern recognition of rolling bearings. Concretely, the extracted fault features based on IMSDE are divided randomly into the training dataset and the testing dataset, where the training datasets are adopted to train the MRVM model and the testing datasets are imported into the well-trained MRVM model for automatically identifying different fault patterns of rolling bearings. The introduction of IMSDE theory can be referred to Appendix A.

### 2.4. Flowchart of the Proposed Method

**Figure 8:**
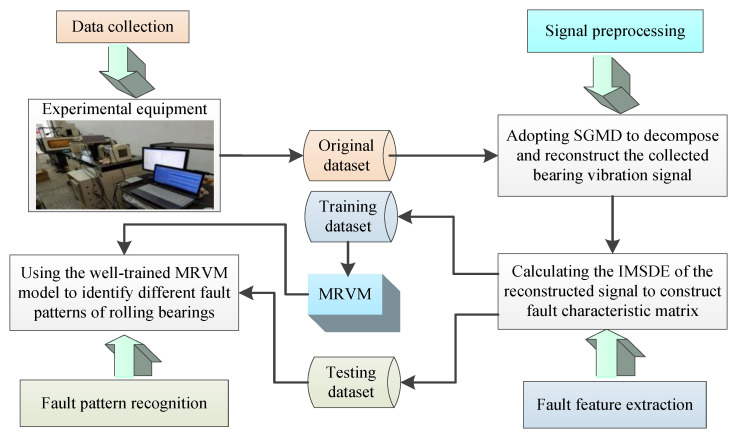
Flowchart of the proposed method for bearing fault identification.

Figure 8 shows the overall flowchart of the proposed method. The general process of the proposed method mainly consists of two parts: the training part and the testing part. Specifically, the collected original bearing dataset is firstly decomposed and reconstructed by SGMD, which is aimed at removing the noise and obtaining the reconstructed bearing dataset. Then, calculating IMSDE of the reconstructed bearing dataset to obtain fault characteristic matrix, where the extracted fault features are randomly divided into the training dataset and testing dataset. That is, in the training part, the training dataset is used to train the MRVM model. However, in the testing part, the testing dataset is fed into the well-trained MRVM model to identify different fault patterns of bearings.

## 3. Experimental Verification

### 3.1. Experimental Setup and Data Description

**Figure 9:**
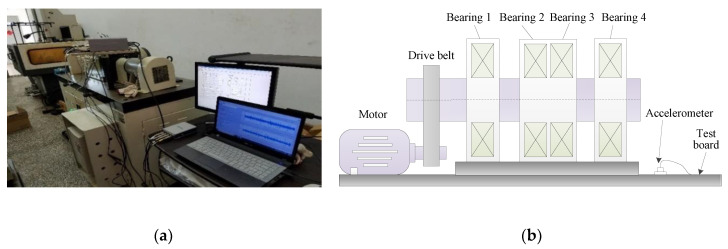
(**a**) Photo of experimental equipment and (**b**) its corresponding structure diagram.

**Figure 10:**
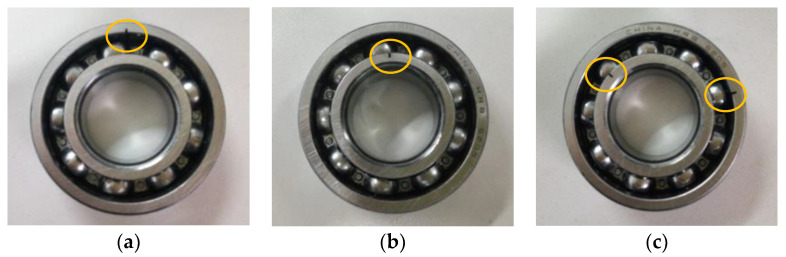
Photo of the faulty bearing: (**a**) outer race fault (ORF), (**b**) inner race fault (IRF) and (**c**) outer-inner race compound fault (OIRF).

**Figure 11:**
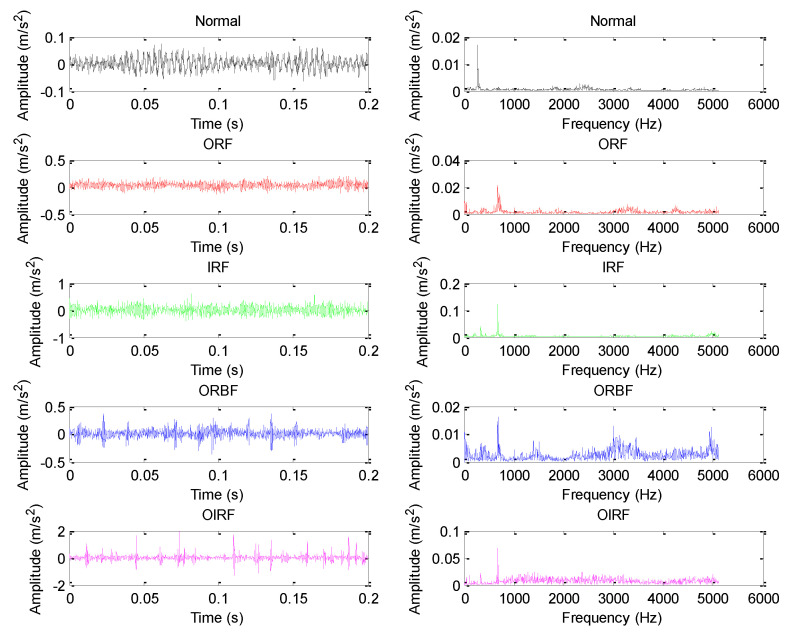
Waveform and spectrum of different bearing vibration signals.

**Figure 12:**
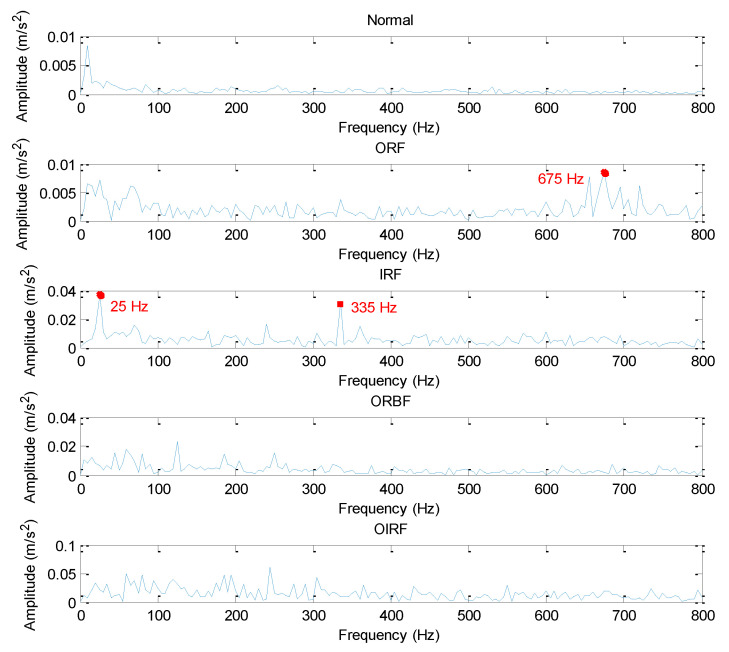
Envelope spectrum of different bearing vibration signals.

The verification of efficacy of our proposed approach is conducted on experimental platform from Research Center of Condition Monitoring and Fault Diagnosis (RCCMFD), Southeast University. Figure 9a,b depict respectively the photograph and schematic drawing of the experimental equipment, which is mainly composed of loading equipment, bearing test module, driving system, electrical control system and computer monitoring equipment. In this experiment, through using spark machining, we simulate four groups of bearing faults (its size is 0.5 mm in depth and 0.1 mm in width), which is, respectively, called outer race fault (ORF), inner race fault (IRF), outer-inner race compound fault (OIRF), and outer race-ball compound fault (ORBF). Figure 10 shows the faulty bearing containing different fault patterns. Table 2 displays the detailed specification of bearing. Motor speed during the experiment is stable at 1050 rpm, and the sampling frequency is 10,240 Hz. Table 3 gives the defect frequencies of bearing. To obtain the weak bearing vibration data, the PCB accelerometer with a sensitivity of 100 mV/g was mounted on one position deviate from testing bearing block to collect different bearing vibration data. For each health conditions, a total of 60 data samples were collected with the length of 2048 points. That is, there are 300 data samples in total, where 150 samples were randomly selected for training dataset and the remainder is regarded as the testing dataset. Table 4 lists the specific information of bearing dataset. Figure 11 plots waveform and spectrum of bearing vibration data under different health conditions. As seen from Figure 11, except for normal state, waveform and spectrum of different bearing fault patterns have certain similarity, which indicates that bearing fault patterns cannot be accurately judged by the direct observation of waveform and spectrum. Figure 12 shows envelope spectrum of different bearing vibration signals. Note that the envelope spectrum $S_{e}(t)$ of the given signal *s*(*t*) with zero mean can be defined by Equation (3), which is intuitively understood as the discrete Fourier transform of the envelope signal. Seen from Figure 12, bearing defect frequencies are almost invisible, and there are some interference ingredients (e.g., 25 Hz, 335 Hz and 675 Hz) in envelope spectrum, but it is important to note here that the envelope spectrum of Figure 12 will show bearing defect frequencies if the sample length used in this experiment is set to be longer. Hence, the experiment analysis under the longer sample length is regarded as our future focus work. In other words, this experiment mainly focuses on bearing fault identification under small sample length. However, whether the small sample length or large sample length, it is valuable to adopt a useful approach to extract automatically bearing fault features and solve five-categories identification problem.

(3) $$\left\{ \begin{array}{l} s_{e}(t)=\left|s(t)+j\cdot \mathrm{Hilbert}[s(t)]\right|\\ S_{e}(f)=\mathrm{DFT}[s_{e}(t)] \end{array}\right.$$

where *s*(*t*) is the given signal, $s_{e}(t)$ is the envelope signal of *s*(*t*), Hilbert[·] denotes the Hilbert transform operator, and DFT[·] represents the discrete Fourier transform operator. More details about the envelope spectrum can be also referred to the literature [35,36].

### 3.2. The Proposed Method Analysis

**Figure 13:**
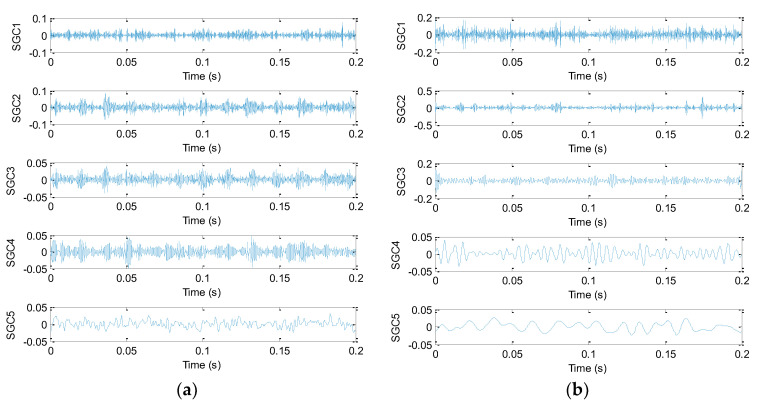
Decomposition results obtained by SGMD for bearing vibraion signal: (**a**) ORF and (**b**) IRF.

**Figure 14:**
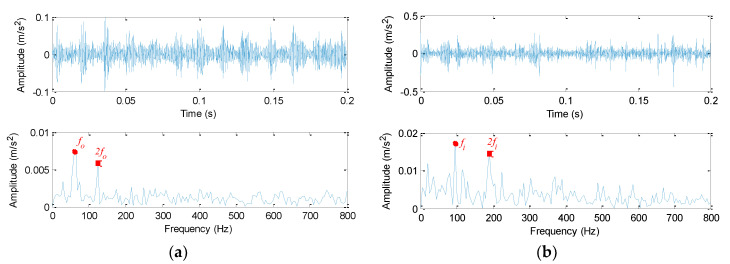
Waveform of reconstructed bearing vibraion signal and its envelope spectrum: (**a**) ORF and (**b**) IRF.

**Figure 15:**
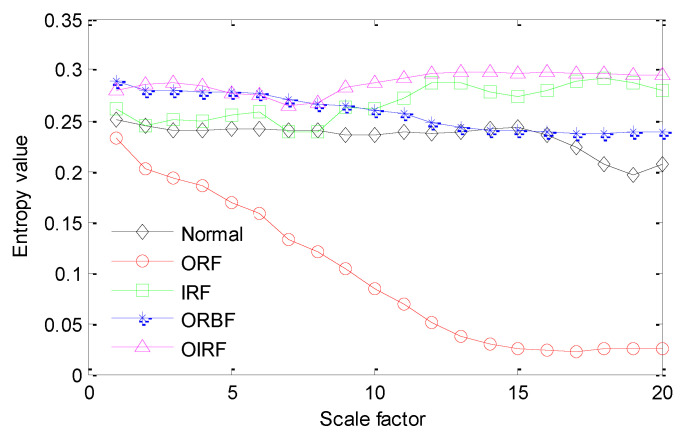
Calculation result of IMSDE for one data sample of each category.

**Figure 16:**
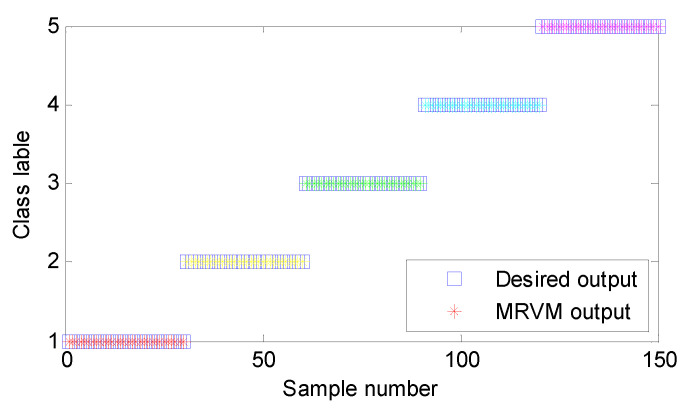
Identification result of the proposed method in the first trial.

The proposed method is adopted to analyze bearing vibration data. Firstly, SGMD is used to preprocess the original bearing vibration data and obtain five SGC components, where the first two SGC components with higher correlation coefficients are used to reconstruct the original signal and remove noise interference. Take the ORF and IRF signal as an example, Figure 13a,b show decomposition results obtained by SGMD for the ORF and IRF signal, respectively. Figure 14a shows waveform and envelope spectrum of the reconstructed ORF signal, while Figure 14b shows waveform and envelope spectrum of the reconstructed IRF signal. Seen from Figure 14, after SGMD reconstruction, the noise interference of the original signal can be removed greatly. Besides, the OR and IR defect frequencies (*f*_o_ and *f*_i_) and their frequency doublings (2 *f*_o_ and 2 *f*_i_) can be extracted obviously in envelope spectrum of the reconstructed signal, which shows the efficacy of SGMD in signal preprocessing. Secondly, IMSDE of each reconstructed signal is calculated to construct a multi-dimensional vector with 300 rows and 20 columns, where the important parameters of IMSDE is optimized as τ = 20, *m* = 3, λ = 1 and ε = 12 by using BA. Figure 15 shows IMSDE value of different fault patterns for one data sample. As seen from Figure 15, the entropy value of different fault patterns under same scale factor is different. Besides, entropy value of same fault patterns under different scale factor is also different. This indicates that bearing fault features with significant differences can be excavated efficiently by using IMSDE. Finally, the extracted multi-dimensional features are randomly divided into the training dataset and the testing dataset. Specifically, the training/testing dataset percentage is 1:1, where the training dataset with 150 rows and 20 columns are adopted to train the MRVM model and the remainder testing dataset are fed into the well-trained MRVM model for intelligent fault identification of rolling bearings. Figure 16 shows detailed identification result of different bearing fault patterns in the first trial. Seen from Figure 16, bearing fault patterns can be fully identified and the fault identification rate reaches 100%, which implies that the proposed method is promising for bearing intelligent fault identification.

To avoid the contingency of diagnostic results, four trials of the proposed method under different number of symbols are conducted. Table 5 lists the detailed identification results of our method. As shown in Table 5, average identification accuracy (99.83%) of the proposed method is maximum when the number of symbols is selected as 12, which verifies the effectiveness of BA-based optimized IMDSE used in the proposed algorithm. That is, it is effective for improving the identification accuracy by using BA algorithm to optimize the parameters of IMDSE. Besides, as the number of symbols increases, average computation time of the proposed algorithm will be increased to some extent: that is, the larger the number of symbols, the longer the calculation time of the proposed method. Hence, in our future work, we will focus on improving the computational efficiency of the proposed method.

### 3.3. Comparison among Various Methods

Comparison among various methods are further conducted to demonstrate the efficacy and advantages of the proposed approach. On the one hand, to show the effectiveness of SGMD in signal preprocessing, IMSDE containing different preprocessors is integrated with various classifiers (e.g., MRVM, BPNN, SVM, ELM and KNN) to analyze the same experimental data. Table 6 lists main parameter setting of various classifiers. More details of various classifiers are available at literature [16]. Table 7 gives the identification results of combining various classifiers with IMSDE containing different preprocessors. Seen from Table 7, average testing accuracy of combining SGMD-IMSDE with various classifiers is 99.33% (745/750), which is higher than that of combining other three methods (EEMD-IMSDE, EWT-IMSDE and EITD-IMSDE) with various classifiers, which is 94.26% (707/750), 96.53% (724/750) and 93.20% (699/750), respectively. This indicates that SGMD is more efficient than other signal preprocessors (EEMD, EWT and EITD). That is, SGMD is more suitable for signal noise reduction. Besides, average testing accuracy of combining MRVM with various methods (SGMD-IMSDE, EEMD-IMSDE, EWT-IMSDE and EITD-IMSDE) is 96.83% (581/600), which is bigger than that of combining other classifiers with various methods, which is 95.16% (571/600), 96.00% (576/600), 95.67% (574/600) and 95.50% (573/600), respectively. This means that MRVM is more helpful in bearing fault identification than other classifiers (BPNN, SVM, ELM and KNN).

On the other hand, to illustrate the efficacy of IMSDE in feature extraction, SGMD containing different feature extractors is integrated with various classifiers (e.g., MRVM, BPNN, SVM, ELM and KNN) to process the same experimental data. Note that, to ensure the fairness of comparison, key parameters of various feature extractors (IMSDE, MSDE, MSE and MPE) are determined by BA method. Table 8 lists the detailed identification results of different combination methods. Seen from Table 8, average accuracy of combining SGMD-IMSDE with various classifiers is 99.33% (745/750), which is higher than that of combining other three methods (SGMD-MSDE, SGMD-MSE and SGMD-MPE) with various classifiers, which are 97.73% (733/750), 91.73% (688/750) and 95.20% (714/750), respectively. This results further illustrate the superiority of IMSDE in feature extraction. In other words, IMSDE is a more promising feature extractor than other available feature extractors (MSDE, MSE and MPE). Furthermore, average accuracy of combining MRVM with various methods (SGMD-IMSDE, SGMD-MSDE, SGMD-MSE and SGMD-MPE) is 96.83% (581/600), which is also greater than those in combining other classifiers with various methods, which are 95.50% (573/600), 95.83% (575/600), 96.00% (576/600) and 95.83% (575/600), respectively. The analysis results further validate that MRVM is more efficient than other classifiers.

Overall speaking, the validity and superiority of the proposed method is highlighted through the comparative analysis. There are several reasons for causing this result. Firstly, SGMD is adopted to preprocess the original bearing vibration signal, which can obtain more effective noise reduction performance than other signal decomposition methods (EEMD, EWT and EITD). Secondly, due to the improvement of coarse-grained process and the application of entropy average strategy, IMSDE can achieve better feature extraction effect than other feature extractors (MSDE, MSE and MPE). Thirdly, due to the performance of MRVM is less affected by the parameters, MRVM is more suitable for intelligent fault identification than other classifiers (BPNN, SVM, ELM and KNN).

## 4. Conclusions

This paper proposes an intelligent fault diagnosis approach for rolling bearing based on SGMD, IMSDE and MRVM. The biggest advantage of the fusion technology is that the accuracy of bearing fault diagnosis can be greatly improved. Meanwhile, IMSDE can improve the feature extraction performance of MSDE by modifying coarse-grained procedure and applying BA-based parameter optimizer. The effectiveness of the integrated algorithm is verified by experimental analysis. Besides, the proposed method is very competitive compared to some related approaches involved in this paper. Main contributions of this paper can be summarized into three aspects:

(1) SGMD is applied for signal preprocessing, which can remove noise interference hidden in the original bearing vibration signal and reveal fault symptoms.
(2) The BA-based optimized IMSDE is proposed for fault feature extraction, which can determine automatically its important parameters and avoid the disadvantage of information loss of MSDE.
(3) MRVM classifier is introduced for fault pattern recognition, which can achieve better multi-classification performance than other involved classifiers.

Experimental results and comparative analysis show that the proposed algorithm is effective in the identification of bearing fault categories. However, efficacy of the proposed algorithm for the recognition of bearing fault severity in whole-life stage are unknown. Hence, in our future work, we will focus on how to investigate the recognition performance of the proposed method in bearing fault severities. Besides, the improvement of computational efficiency of the proposed algorithm is also our future research emphasis. Another saying is that if data length of the adopted samples is long enough, other similar methods (e.g., EEMD-IMSDE, EWT-IMSDE and EITD-IMSDE) may get the same good identification results with less processing. Hence, the experiment analysis under the lager sample length is regarded as our future key emphasis in work.

## Figures and Tables

**Table 1:** Decomposition index comparison among different methods.

Methods	$\rho_{1}$	$\rho_{2}$	$\rho_{3}$	RMSE	Computing Time (s)
SGMD	0.9987	0.9998	0.9942	0.0741	1.1749
EEMD	0.4901	0.8215	0.9905	0.0985	3.2671
EWT	0.7424	0.0147	0.0207	2.7231	1.0938
EITD	0.8435	0.7298	0.1721	0.1965	1.0142

**Table 2:** Parameters of rolling bearing.

Bearing Type	Ball Diameter (mm)	Pitch Diameter (mm)	Number of Balls	Contact Angle (°)
HRB6205	7.94	39.04	9	0

**Table 3:** Defect frequencies of rolling bearing (Hz).

Rotation Frequency *f_r_*	Inner Race Fault *f_i_*	Outer Race Fault *f_o_*	Ball Fault *f_b_*	Cage Fault *f_c_*
17.5	94.76	62.73	41.24	6.97

**Table 4:** Description of experimental dataset.

Fault Type	Number of Training Samples	Number of Testing Samples	Class Labels
Normal	30	30	1
ORF	30	30	2
IRF	30	30	3
OIRF	30	30	4
ORBF	30	30	5

**Table 5:** Identification results of the proposed method in four trials.

Number of Symbols	Diagnosis accuracy and CPU Time Obtained Using Our Method for Four Trials				Average Accuracy and CPU Time
	1	2	3	4	
ε = 3	95.33%/18.25 s	94.67%/18.83 s	94.67%/17.96 s	94.00%/17.07 s	94.67%/18.02 s
ε = 6	98.00%/26.72 s	97.33%/27.13 s	97.33%/26.94 s	98.00%/25.08 s	97.67%/26.46 s
ε = 12	100%/33.89 s	100%/34.13 s	99.33%/33.52 s	100%/34.38 s	99.83%/33.98 s
ε = 18	96.67%/41.33 s	96.00%/42.05 s	97.33%/41.26.s	97.33%/41.47 s	96.83%/41.52 s

**Table 6:** Main parameter setting of various classifiers.

Different Classifiers	Main Parameter Setting
MRVM	Kernel parameters of radial basis function *g* = 0.05
BPNN	Number of hidden nodes *N* = 20, the training number *I* = 500, the learning rate *σ* = 0.1, and the training error *e* = 0.001
SVM	Penalty factor *c* = 1, kernel parameters of radial basis function *g* = 0.05
ELM	Number of hidden neurons *N* = 20
KNN	Number of neighbors is set as 3

**Table 7:** Diagnosis results of combining various classifiers with IMSDE containing different preprocessors.

Different Classifiers	Testing Accuracy Obtained Using IMSDE with Different Preprocessors				Average Accuracy
	SGMD-IMSDE	EEMD-IMSDE	EWT-IMSDE	EITD-IMSDE	
MRVM	100% (150/150)	95.33% (143/150)	97.33% (146/150)	94.67% (142/150)	96.83% (581/600)
BPNN	98.67% (148/150)	94.00% (140/150)	96.00% (144/150)	92.67% (139/150)	95.16% (571/600)
SVM	99.33% (149/150)	94.67% (142/150)	96.67% (145/150)	94.00% (140/150)	96.00% (576/600)
ELM	99.33% (149/150)	94.00% (140/150)	96.67% (145/150)	93.33% (140/150)	95.67% (574/600)
KNN	99.33% (149/150)	94.67% (142/150)	96.00% (144/150)	92.00% (138/150)	95.50% (573/600)
Average accuracy	99.33% (745/750)	94.26% (707/750)	96.53% (724/750)	93.20% (699/750)	——

**Table 8:** Diagnosis results of combining various classifiers with SGMD containing different feature extractors.

Different Classifiers	Testing Accuracy Obtained Using SGMD with Different Feature Extractors				Average Accuracy
	SGMD-IMSDE	SGMD-MSDE	SGMD-MSE	SGMD-MPE	
MRVM	100% (150/150)	98.67% (148/150)	92.67% (139/150)	96.00% (144/150)	96.83% (581/600)
BPNN	98.67% (148/150)	97.33% (146/150)	91.33% (137/150)	94.67% (142/150)	95.50% (573/600)
SVM	99.33% (149/150)	98.00% (147/150)	91.33% (137/150)	94.67% (142/150)	95.83% (575/600)
ELM	99.33% (149/150)	97.33% (146/150)	92.00% (138/150)	95.33% (143/150)	96.00% (576/600)
KNN	99.33% (149/150)	97.33% (146/150)	91.33% (137/150)	95.33% (143/150)	95.83% (575/600)
Average accuracy	99.33% (745/750)	97.73% (733/750)	91.73% (688/750)	95.20% (714/750)	——

**Figure A1:**
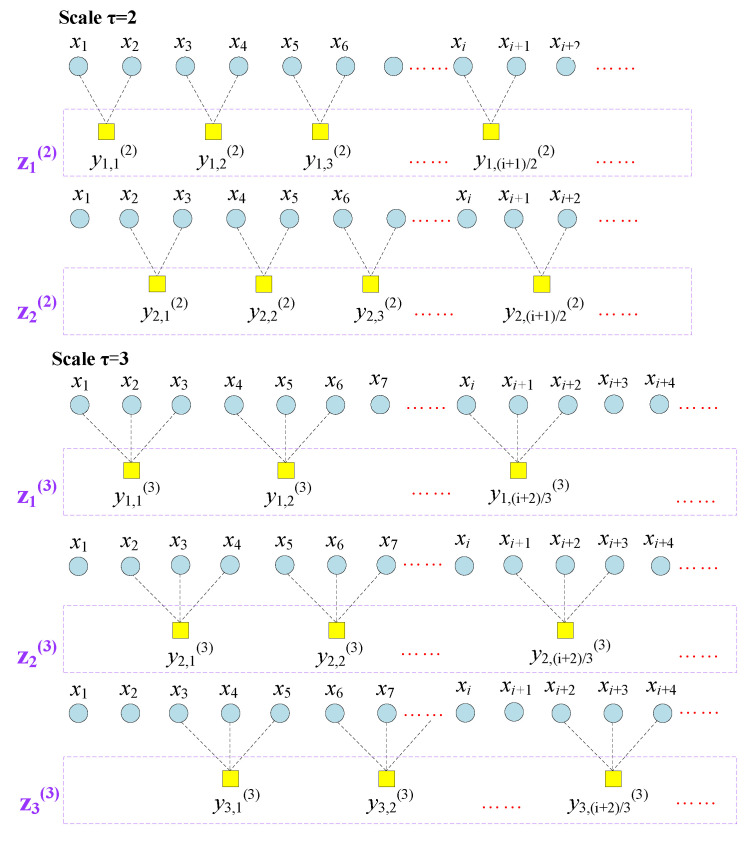
The improved coarse-grained procedure with data slipping operation.

**Figure A2:**
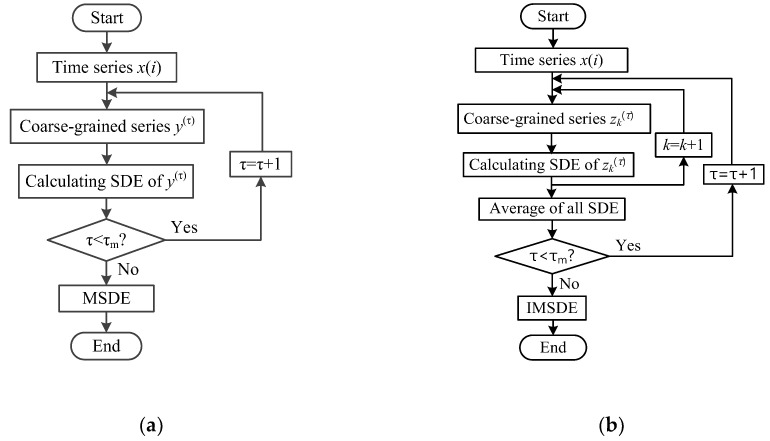
Flowchart comparison between MSDE and IMSDE: (**a**) MSDE and (**b**) IMSDE.

## Supplementary Materials

The following are available online at https://www.mdpi.com/1424-8220/20/15/4352/s1, MATLAB code.

## Appendix A. Related Theory

### Appendix A.1. SGMD

SGMD is a new adaptive signal decomposition method, which mainly consists of three steps (i.e., phase space reconstruction, QR decomposition of symplectic orthogonal matrix and diagonal average operation). The general steps of SGMD are summarized as follows:

(1) Phase space reconstruction. Supposing that there is a one-dimensional discrete original signal $x=(x_{1},x_{2},\ldots ,x_{n})$, then we have
  (A1) $$X=\left[\begin{array}{cccc} x_{1} & x_{1+\tau } & \cdots & x_{1+(k-1)\tau }\\ x_{2} & x_{2+\tau } & \cdots & x_{2+(k-1)\tau }\\ \vdots & \vdots & \vdots & \vdots \\ x_{m} & x_{m+\tau } & \cdots & x_{m+(k-1)\tau } \end{array}\right]$$
  where *k* is the embedding dimension, τ is the time delay and m=n−(k−1)τ. Due to the two parameters (i.e., embedding dimension *k* and time delay τ) have certain influence on the reconstruction results, so we need to choose them carefully. In this paper, considering the influence of time delay on the analysis results is much smaller than the embedding dimension. Hence, in this paper, time delay is empirically selected as τ = 1, whereas the embedding dimension *k* is determined adaptively by calculating power spectral density (PSD) based on the idea of literature [37]. Specifically, PSD of the original signal *x* is firstly calculated, then the frequency $f_{\max}$ corresponding to the maximum peak in PSD is estimated, if the normalized frequency $f_{\max}/F_{s}$ is less than the given threshold 0.001, the embedding dimension *k* is set as *n*/3, where *n* is the data length. Otherwise, the embedding dimension is set as $k=1.2\times (F_{s}/f_{\max})$, where *F_s_* is the sampling frequency.
(2) QR decomposition of symplectic orthogonal matrix. Assuming that the covariance symmetric matrix $A=X^{T}X$, the Hamilton matrix M can be constructed as
  (A2) $$M=\left[\begin{array}{cc} A^{T} & 0\\ 0 & -A \end{array}\right]$$
  If $N=M^{2}$, then both *M* and *N* are Hamiltonian matrices. Symplectic orthogonal matrix *Q* can be constructed, such that
  (A3) $$Q^{T}NQ=\left[\begin{array}{cc} B & R\\ 0 & B^{T} \end{array}\right]$$
  where *B* is the upper triangular matrix and $b_{ij}=0\;(i>j+1)$. Besides, *B* can be obtained by transforming *N* into two symplectic orthogonal matrices, whose eigenvalues are $\lambda_{1}$, $\lambda_{2}$,..., $\lambda_{d}$. According to the property, $\sigma_{1}=\sqrt{\lambda_{i}}$. $Q_{i}$ is the eigenvector of matrix *A* corresponding to $\sigma_{i}$. According to $S_{i}=Q_{i}^{T}X^{T}$ and $Y=Q_{i}S_{i}$, we can get the matrix $X_{i}=Y^{T}$. The original phase space reconstruction matrix *X* is composed of *d* components, which can be rewritten as $X=X_{1}+X_{2}+\ldots +X_{d}$.
(3) Diagonal average operation. In this step, the one given components $X_{k}$ (1 ≤ k ≤ d) can be transformed into a time series with the length n, so the original time series amounts to the sum of d time series with the length n. Assuming that $X_{m\times d}={\left(x_{ij}\right)}_{m\times d}$, $d^{*}=\min(m,d)$, $m^{*}=\max(m,d)$, n=m+(d−1)τ.
  (A4) $$y_{k}=\{\begin{array}{ll} \frac{1}{k}\sum_{p=1}^{k}y_{p,k-p+1}^{*} & 1\leq k\leq d^{*}\\ \frac{1}{d}\sum_{p=1}^{d^{*}}y_{p,k-p+1}^{*} & d^{*}\leq k\leq m^{*}\\ \frac{1}{n-k+1}n\sum_{p=k-m^{*}+1}^{n-m^{*}+1}y_{p,k-p+1}^{*} & m^{*}\leq k\leq n \end{array}$$
  According to Equation (A4), the time series $Y_{i}=(y_{1},y_{2},\ldots ,y_{n})$ can be obtained from $X_{i}$, finally the original vibration signal can be divided successively into *d* independent mono-component signals named symplectic geometry component (SGC).

### Appendix A.2. IMSDE

Figure A1 shows the flowchart of the proposed IMSDE. Figure A2 shows the flowchart comparison between MSDE and IMSDE, where $\tau_{m}$ denotes the largest scale factor. Detailed implementation procedure of IMSDE are summarized as follows:

(1) For the scale factor τ of time series {x(i):1≤i≤N}, using Equation (A5) to obtain the coarse-grained time series $z_{k}^{\left(\tau \right)}=\{y_{k,1}{}^{\left(\tau \right)},y_{k,2}{}^{\left(\tau \right)},\cdots \}$. Specifically, in IMSDE, if the scale factor τ = 1, we can acquire one coarse-graining time series $z_{k}^{\left(1\right)}$. If the scale factor τ = 2, we can acquire two coarse-graining time series $z_{k}^{\left(1\right)}$ and $z_{k}^{\left(2\right)}$. However, in MSDE, when the scale factor τ = 2, only one coarse-graining time series $z_{k}^{\left(1\right)}$ is acquired.
  (A5) $$y_{k,j}{}^{\left(\tau \right)}=\frac{\sum_{f=0}^{\tau -1}x_{f+k+\tau (j-1)}}{\tau },\;1\leq j\leq \left\lfloor \frac{N}{\tau }\right\rfloor ,\;1\leq k\leq \tau$$
(2) Calculating the symbolic dynamic entropy (SDE) of each coarse-grained time series $z_{k}^{\left(\tau \right)}(k=1,2,\cdots ,\tau )$, and conducting the average operation of all SDE to obtain the final results of IMSDE (see Equation (A6)).
  (A6) $$\mathrm{IMSDE}(x,\tau ,m,\lambda ,\varepsilon )=\frac{1}{\tau }\sum_{k=1}^{\tau }SDE(z_{k}^{\left(\tau \right)},m,\lambda ,\varepsilon )$$
  where τ represents the scale factor, *m* denotes the embedding dimension, λ means the time delay, ε represents the number of symbols. SDE(·) denotes the operator of symbolic dynamic entropy. Detailed calculation procedure of SDE is available at [14], where the performance of SDE has been proved better than other entropies (e.g., SE and PE) at describing the frequency and amplitude change of signal. Given these facts, theoretically speaking, due to the superiority of SDE in describing the complexity of signal, and the application of data sliding and average operation, the modified coarse-grained procedure can more comprehensively describe signal’s amplitude information at different scales, so IMSDE can obtain more abundant fault characteristic information than MSDE and some existing multiscale entropies (e.g., MSE and MPE). Note that the modified coarse-grained procedure in IMSDE can also be found in literature [38,39]. If one wants to implement this algorithm, its MATLAB code are available from the corresponding author upon request, or you can also download it from the Supplementary Materials.

### Appendix A.3. MRVM

In MRVM, the probability of fault categories can be obtained by adopting hierarchical Bayesian structure and introducing multiple probability likelihood function. Theory of MRVM is summarized as follows:

Supposing that the training sample set is $X=\{x_{i},{t_{i}\}}_{i=1}^{N}$, where t∈{1,2,…,C} is the category label of data sample. When the kernel function is selected, the training kernel function set $K={\left(k_{i},\ldots ,k_{N}\right)}^{T}$, $K\in R^{N\times N}$ can be obtained, where $k_{n}$ represents the similarity between the *n*-th sample of the training dataset and other samples. By introducing the auxiliary regression target $Y\in R^{L\times N}$ and the weight parameter $W\in R^{N\times L}$, the standard noise regression model can be obtained as:

(A7) $$y_{nl}|w_{l},k_{n}\sim N_{ynl}(k_{n}w_{l},l)$$

where $y_{nl}$ is the element of the *n*-th row and *l*-th column of *Y*; $w_{l}$ is the *l*-th column of w; $N_{x}(m,v)$ denotes the normal distribution with the mean value of *m* and the variance of *v*.

Enter the polynomial (5) to convert the regression target into the category label.

(A8) $$t_{n}=i,y_{ni}>y_{nj},\forall j\neq i$$

To ensure the sparsity of model, the standard normal prior distribution with mean value of 0 and variance of $a_{nl}^{-1}$ is introduced for the weight vector. The matrix composed of prior parameter $a_{nc}$ is recorded as $A\in R^{N\times L}$, which obeys gamma distribution with the super parameter $a_{nl}^{-1}$. Hence, the posterior probability can be deduced as:

(A9) $$P(W|Y)\propto P(Y|W)P(W|A)\propto \prod_{c=1}^{c}N({\left(KK^{T}+A_{c}\right)}^{-1}Ky_{c}^{T}{\left(KK^{T}+A_{c}\right)}^{-1})$$

where $A_{c}$ is the diagonal matrix derived from *c* column of matrix *A*. From the estimation of maximum posterior probability, we can get:

(A10) $$\overset{\wedge }{W}=\arg\;\max_{w}P(W|Y,A,K)$$

Therefore, according to the known categories, the updating method of weight of maximum posterior estimation is expressed as:

(A11) $$\overset{\wedge }{w_{c}}={\left(KK^{T}+A_{c}\right)}^{-1}Ky_{c}^{T}$$

The posterior probability distribution of prior parameters of the weight vector is calculated by:

(A12) $$P(A|W)\propto P\pi (\left|W\right|A)P(A|\tau ,v)\propto \prod_{c=1}^{C}\prod_{n=1}^{N}G(\tau +\frac{1}{2},\frac{w_{nc}^{2}+2v}{2})$$

Finally, according to Equation (A12), probability of the unknown samples belonging to different fault categories can be obtained. The category with the highest probability corresponding to the unknown sample is recognized as the fault category.
